# Supplementary material for: Evaluating citizen science outreach: A case-study with The Peregrine Fund’s American Kestrel Partnership
Source: PLoS One. 2021 Mar 30;16(3):e0248948. doi: 10.1371/journal.pone.0248948 (PMC8009395; doi:10.1371/journal.pone.0248948)
Supplement: S1 Appendix — Text for full survey instrument used to evaluate outreach strategy for The Peregrine Fund’s American Kestrel Partnership. Text of survey was entered into the online survey software platform, Qualtrics, such that a skip logic questioning design was employed. For example, a “no” answer on Q1 would bring Q12 up as the next question that respondent sees. (PDF) [file pone.0248948.s001.pdf]

S1 Appendix: Text for full survey instrument used to evaluate outreach strategy for The Peregrine Fund's American Kestrel Partnership. Text of survey was entered into the online survey software platform, Qualtrics, such that a skip logic questioning design was employed. For example, a "no" answer on Q1 would bring Q12 up as the next question that respondent sees.

introductory text: The American Kestrel Partnership (AKP) is partnering with Boise State University to conduct a survey of people who have expressed an interest in American Kestrels. You are receiving this survey because you either registered as a partner or signed up for email notifications on the AKP website or The Peregrine Fund's KestrelCam. Your participation will help AKP better understand you, our greatest supporters, and help us improve our education and outreach programs. Please note that your participation in this study is completely voluntary and all responses will be completely anonymous—no identifying information will be collected.

The survey will take approximately 15 minutes to complete. Thank you, in advance, for your time. Your input is very valuable.

If you have any questions or concerns about your participation in this study, feel free to contact the principal investigators, Dr. Greg Hill (greghill@boisestate.edu) or Dr. Matthew May (matthewmay1@boisestate.edu).

You must be at least 18 years of age to complete the survey.

consent Do you certify that you are at least 18 years old and consent to participate in this survey?
- ❍ Yes, I consent to participate
- ❍ No, I do not consent to participate

Condition: No, I do not consent to par... Is Selected. Skip To: End of Survey.

Q1 Do you currently have a kestrel box on your property or that you monitor?
- ❍ Yes
- ❍ No

Display This Question:
If Do you currently have a kestrel box on your property or that you monitor? Yes Is Selected

Q2 Why did you decide to install a kestrel box? (Check all that apply)
- ❑ It seemed like a fun activity to do with my family and/or community
- ❑ I like participating in citizen scientist projects
- ❑ I like bird watching/having birds in my yard
- ❑ I wanted to help increase the kestrel population in my area
- ❑ I wanted to partner with AKP

Display This Question:
If Do you currently have a kestrel box on your property or that you monitor? Yes Is Selected

Q3 How long have you had or monitored your kestrel box(es)? (If you have multiple, use your first box to answer this question)

- ❍ Less than a year
- ❍ 1- 5 years
- ❍ 5- 10 years
- ❍ 10 plus years

Display This Question:
If Do you currently have a kestrel box on your property or that you monitor? Yes Is Selected

Q4 If you own or monitor multiple boxes, please tell us how many:

- ❍ I only have one box
- ❍ 2
- ❍ 3-10
- ❍ 11-30
- ❍ More than 30

Display This Question:
If Do you currently have a kestrel box on your property or that you monitor? Yes Is Selected

Q5 Have you registered your box(es) with AKP's nest box database?

- ❍ Yes
- ❍ No

Display This Question:
If Do you currently have a kestrel box on your property or that you monitor? Yes Is Selected
And Have you registered your box(es) with AKP's nest box database? No Is Selected

Q5a Please indicate why your nest boxes are not registered with the AKP nest box database. (Check all that apply)

- ❑ I didn't know about the AKP nest box database
- ❑ I enter my data into a different organization's database
- ❑ The website is too complicated
- ❑ Other ____________________

Display This Question:

If Do you currently have a kestrel box on your property or that you monitor? Yes Is Selected

Q6 Did someone from AKP – either a volunteer or employee – help you build or set up your box in person?

❍ Yes
❍ No

Display This Question:

If Do you currently have a kestrel box on your property or that you monitor? Yes Is Selected

Q7 How often do you monitor your box(s)?

❍ Every week during breeding season
❍ Every two weeks during breeding season
❍ Twice per breeding season (once when eggs and once again within 30 days)
❍ Until I see eggs and then I leave them alone
❍ Once a year
❍ Never
❍ Other ____________________

Display This Question:

If How often do you monitor your box(s)? Never Is Selected

And Do you currently have a kestrel box on your property or that you monitor? Yes Is Selected

Q7a Please indicate why your nest boxes are not monitored. (Check all that apply)

❑ I don't have time
❑ I don't know how
❑ It is too physically demanding
❑ I don't want to disturb the birds
❑ I didn't know someone wanted me to
❑ Other ____________________

Display This Question:

If Do you currently have a kestrel box on your property or that you monitor? Yes Is Selected

Q8 Would you consider using a smartphone app for data collection and entry while monitoring your boxes that would upload your data directly to the AKP website?

❍ Yes
❍ No

Display This Question:

If How often do you monitor your box(s)? Never Is Not Selected

And Do you currently have a kestrel box on your property or that you monitor? Yes Is Selected

Q9 Do you follow AKP’s recommendations for monitoring boxes when checking your box(es)? These can be found under "partnership documents" on the AKP website.

❍ Yes

❍ No

Display This Question:

If Do you follow AKP’s recommendations for monitoring boxes when checking your box(es)? These can be found under "partnership documents" on the AKP website. No Is Selected

And Do you currently have a kestrel box on your property or that you monitor? Yes Is Selected

Q9a Please indicate why you don't follow AKP's monitoring recommendations.

❍ I don’t follow any protocol, I just check them when I can

❍ I didn’t know AKP had a recommended protocol

❍ It is too much work to follow AKP’s protocol

❍ Other ____________________

Display This Question:

If Do you currently have a kestrel box on your property or that you monitor? Yes Is Selected

Q10 Do you input your monitoring data into the AKP database?

❍ Yes

❍ No

Display This Question:

If Do you input your monitoring data into the AKP database? No Is Selected

And Do you currently have a kestrel box on your property or that you monitor? Yes Is Selected

Q10a Please indicate why you don't input your monitoring data into the AKP database.

❍ I didn’t know AKP had a database

❍ I enter my data into a different organization’s database

❍ I don’t have time

❍ The website is too complicated

❍ Other ____________________

Display This Question:

If Do you currently have a kestrel box on your property or that you monitor? No Is Selected

Q11 Are you considering putting up a kestrel box in the future?

- ❍ Yes
- ❍ No

Display This Question:

If Do you currently have a kestrel box on your property or that you monitor? No Is Selected

Q12 What factors might convince you to commit to put up and monitor a box and submit your data to AKP? (Check all that apply)

- ❑ If someone from AKP helped me put up the box
- ❑ If I had access to information on all the steps involved
- ❑ If it were easy to monitor and upload data
- ❑ If kestrel monitoring required a small time commitment
- ❑ No convincing needed, I will commit to monitoring a box and submitting my data next breeding season
- ❑ I probably cannot be convinced to do this
- ❑ Other ____________________

Q13 Do you feel that you have learned about American Kestrel biology and/or efforts by The Peregrine Fund to understand the decline of the American Kestrel?

- ❍ Yes
- ❍ No

Q14 What do you understand to be the major cause of kestrel declines in North America?

- ❍ Pesticide use
- ❍ Cooper’s Hawk predation
- ❍ European Starling competition
- ❍ Cavity loss
- ❍ Habitat loss (other than cavities)
- ❍ Climate change
- ❍ I don’t know
- ❍ I didn’t know they were in decline
- ❍ Other ____________________

Q15 Do you think installing nest boxes will reverse the kestrel decline?

- ❍ Yes
- ❍ I don't know
- ❍ No
- ❍ Other ____________________

Q16 How did you find out about AKP? (Check all that apply)

- ❑ A friend or family member told me about it
- ❑ Through The Peregrine Fund (website, communications, in person, etc.)
- ❑ Through someone associated with AKP (a volunteer or employee)
- ❑ I read about it on social media
- ❑ Through another birding/conservation organization or club
- ❑ Other ____________________

Q17 How often do you access the AKP website?

- ❍ Weekly
- ❍ Monthly
- ❍ Every six months
- ❍ Once a year
- ❍ Never
- ❍ Other ____________________

Q18 Please rank each type of communication from AKP by importance (5 being most important, 1 being least)

	1	2	3	4	5
Receiving quarterly newsletters via email	❍	❍	❍	❍	❍
Social media outreach such as Facebook	❍	❍	❍	❍	❍
Reading or posting comments on the AKP website	❍	❍	❍	❍	❍
Accessing the KestrelCam	❍	❍	❍	❍	❍
In person interaction (saw a talk, met staff at an event, personal visit, etc.)	❍	❍	❍	❍	❍

Q19 Are you involved with other birding/conservation organizations?

❍ Yes

❍ No

Condition: No Is Selected. Skip To: How have you interacted with AKP? (Ch....

Q19a With which other birding organizations are you involved? (Please list)

Q20 How have you interacted with AKP? (Check all that apply)

❑ Registered as a partner on the AKP website
❑ Watched the KestrelCam
❑ Visited the World Center for Birds of Prey
❑ Registered for email updates (AKP, KestrelCam, etc.)
❑ Follow on social media (AKP Facebook, AKP Twitter)
❑ Purchased AKP T-shirt
❑ Donated to AKP
❑ Donated to The Peregrine Fund
❑ Participated in Adopt-A-Box
❑ Attended a presentation about AKP
❑ Other ____________________

Q21 What is your age?

❍ Under 30
❍ 31-42
❍ 43-54
❍ 55-64
❍ Over 65
❍ I prefer not to answer

Q22 What best describes you?

❍ Male
❍ Female
❍ Other
❍ I prefer not to answer

Q23 What is your highest attained education level?

❍ High school/GED
❍ Some college
❍ Undergraduate
❍ Graduate
❍ I prefer not to answer

Q24 Is there anything else you'd like to tell us?
