# Supplementary material for: Evaluating citizen science outreach: A case-study with The Peregrine Fund’s American Kestrel Partnership
Source: PLoS One. 2021 Mar 30;16(3):e0248948. doi: 10.1371/journal.pone.0248948 (PMC8009395; doi:10.1371/journal.pone.0248948)
Supplement: S1 Table — For each response variable (first column), we present the coefficients (β), standard errors (SE), z values (z), and p values (p) of the predictor variables. (PDF) [file pone.0248948.s003.pdf]

S1 Table. Full and conditional model-averaged estimates from linear (Participation Score) and generalized linear models (Perception of Learning and Agreement with Message). For each response variable (first column), we present the coefficients (β), standard errors (SE), z values (z), and p values (p) of the predictor variables.

		Full				Conditional			
Perception of Learning	Parameter	β	SE	z	p	β	SE	z	p
	Intercept	0.04	0.44	0.09	0.93	0.04	0.44	0.09	0.93
	Discussion Board	0.12	0.10	1.14	0.26	0.17	0.09	1.93	0.05
	KestrelCam	0.12	0.10	1.23	0.22	0.16	0.08	2.05	0.04
	Newsletter	0.12	0.10	1.21	0.23	0.16	0.08	2.03	0.04
	Participation Score	0.24	0.09	2.72	0.01	0.25	0.08	2.94	0.00
	Social Media	-0.01	0.05	0.25	0.80	-0.04	0.08	0.50	0.62
Participation Score	Parameter	β	SE	z	p	β	SE	z	p
	Intercept	2.13	0.28	7.53	<2e-16	2.13	0.28	7.53	<2e-16
	Discussion Board	0.06	0.06	0.97	0.33	0.09	0.05	1.77	0.08
	KestrelCam	-0.36	0.05	7.22	<2e-16	-0.36	0.05	7.22	<2e-16
	Newsletter	0.07	0.06	1.09	0.28	0.10	0.05	1.91	0.06
	Social Media	0.00	0.03	0.10	0.92	-0.01	0.05	0.19	0.85
Agreement with Message	Parameter	β	SE	z	p	β	SE	z	p
	Intercept	1.17	0.36	3.22	0.00	1.17	0.36	3.22	0.00
	KestrelCam	-0.09	0.08	1.08	0.28	-0.12	0.07	1.88	0.06
	Social Media	-0.16	0.07	2.32	0.02	-0.17	0.06	2.81	0.00

Participation Score	0.05	0.06	0.85	0.40	0.09	0.06	1.60	0.11
Newsletter	-0.01	0.04	0.18	0.86	-0.02	0.07	0.36	0.72
Discussion Board	-0.01	0.04	0.20	0.85	-0.03	0.07	0.38	0.70

5
