# Supplementary material for: Evaluating citizen science outreach: A case-study with The Peregrine Fund’s American Kestrel Partnership
Source: PLoS One. 2021 Mar 30;16(3):e0248948. doi: 10.1371/journal.pone.0248948 (PMC8009395; doi:10.1371/journal.pone.0248948)
Supplement: Attachment — Submitted filename: Response to Reviewers_3.5.21.docx [file pone.0248948.s004.docx]

March 5, 2021

Our responses to each comment or concern are highlight in yellow. Thank you! - Sarah

PLOS ONE Decision: Revision required [PONE-D-20-40981] - [EMID:97d335e60421fdf0]

PONE-D-20-40981
Evaluating Citizen Science Outreach: A case-study with The Peregrine Fund’s American Kestrel Partnership
PLOS ONE

Dear Dr. Schulwitz,

Thank you for submitting your manuscript to PLOS ONE. After careful consideration, we feel that it has merit but does not fully meet PLOS ONE’s publication criteria as it currently stands. Therefore, we invite you to submit a revised version of the manuscript that addresses the points raised during the review process.

Please submit your revised manuscript by March 8th 2021. If you will need more time than this to complete your revisions, please reply to this message or contact the journal office at [plosone@plos.org](mailto:plosone@plos.org). When you're ready to submit your revision, log on to <https://www.editorialmanager.com/pone/> and select the 'Submissions Needing Revision' folder to locate your manuscript file.

Please include the following items when submitting your revised manuscript:

- A rebuttal letter that responds to each point raised by the academic editor and reviewer(s). You should upload this letter as a separate file labeled 'Response to Reviewers'.
- A marked-up copy of your manuscript that highlights changes made to the original version. You should upload this as a separate file labeled 'Revised Manuscript with Track Changes'.
- An unmarked version of your revised paper without tracked changes. You should upload this as a separate file labeled 'Manuscript'.

If you would like to make changes to your financial disclosure, please include your updated statement in your cover letter. Guidelines for resubmitting your figure files are available below the reviewer comments at the end of this letter.

If applicable, we recommend that you deposit your laboratory protocols in [protocols.io](http://protocols.io/) to enhance the reproducibility of your results. Protocols.io assigns your protocol its own identifier (DOI) so that it can be cited independently in the future. For instructions see: <http://journals.plos.org/plosone/s/submission-guidelines#loc-laboratory-protocols>

We look forward to receiving your revised manuscript.

Kind regards,

Daniel de Paiva Silva, Ph.D.
Academic Editor
PLOS ONE

Additional Editor Comments:

Dear Sarah Schulwitz et al.,

Congrats!

After careful considerations from two independente reviewers, they find your manuscript is almost already after you take care of the very minor issues both of them raised. Since the change you need to do it very minor, I will give you one month to complete the changes, but I think you will be able to solve them a lot faster than that. Therefore, please resubmit you contribution with a rebuttal letter up to March 8th 2021.

Best regards and keep safe,
Daniel Silva

Journal requirements:

When submitting your revision, we need you to address these additional requirements.

1. Please ensure that your manuscript meets PLOS ONE's style requirements, including those for file naming. The PLOS ONE style templates can be found at

<https://journals.plos.org/plosone/s/file?id=wjVg/PLOSOne_formatting_sample_main_body.pdf> and

<https://journals.plos.org/plosone/s/file?id=ba62/PLOSOne_formatting_sample_title_authors_affiliations.pdf>

Thank you. We have revised the formatting as described in these two links, including revising the formatting of in-text citations and the references section.

[Note: HTML markup is below. Please do not edit.]

Reviewers' comments:

Reviewer's Responses to Questions

**Comments to the Author**

1. Is the manuscript technically sound, and do the data support the conclusions?

The manuscript must describe a technically sound piece of scientific research with data that supports the conclusions. Experiments must have been conducted rigorously, with appropriate controls, replication, and sample sizes. The conclusions must be drawn appropriately based on the data presented.

Reviewer #1: Yes

Reviewer #2: Yes

2. Has the statistical analysis been performed appropriately and rigorously?

Reviewer #1: Yes

Reviewer #2: I Don't Know

3. Have the authors made all data underlying the findings in their manuscript fully available?

The [PLOS Data policy](http://www.plosone.org/static/policies.action#sharing) requires authors to make all data underlying the findings described in their manuscript fully available without restriction, with rare exception (please refer to the Data Availability Statement in the manuscript PDF file). The data should be provided as part of the manuscript or its supporting information, or deposited to a public repository. For example, in addition to summary statistics, the data points behind means, medians and variance measures should be available. If there are restrictions on publicly sharing data—e.g. participant privacy or use of data from a third party—those must be specified.

Reviewer #1: Yes

Reviewer #2: Yes

4. Is the manuscript presented in an intelligible fashion and written in standard English?

PLOS ONE does not copyedit accepted manuscripts, so the language in submitted articles must be clear, correct, and unambiguous. Any typographical or grammatical errors should be corrected at revision, so please note any specific errors here.

Reviewer #1: Yes

Reviewer #2: Yes

5. Review Comments to the Author

Please use the space provided to explain your answers to the questions above. You may also include additional comments for the author, including concerns about dual publication, research ethics, or publication ethics. (Please upload your review as an attachment if it exceeds 20,000 characters)

Reviewer #1: The paper assesses the usefulness of different communication tools in order to reach the projects goals - which are not merely scientific but comprise also environmental education.
A bit strange in the beginning is the "fundraising". While important for the NGO, that is no scientific or outreach target, and in fact later it is not referred to that target. That should be clarified.

Thanks for catching this. We removed the two mentions of fundraising. Line 28 (in the abstract) and Line 39 (1^st^ paragraph).

Is is very interesting to see how the authors came to their disturbing results: If not properly managed, artificial nesting sites can even harm the kestrels.
The paper honestly show that participants have also different targets and resources to participate. I can completely understand the conclusions.

Thank you for your comments.

Reviewer #2: This is a really timely and intriguing paper that can provide lots of value and insight for groups hoping to mount effective outreach campaigns around citizen science efforts. To me the results were somewhat surprising, but perhaps underscore the decreasing relevance of social media as a source of reliable information for its consumers. There is a lot of time and effort going into social media these days, with groups scrambling to figure out how best to leverage that platform for their own messaging. This paper shows that more direct communication with user communities is more effective, and that's a really important outcome.

Thank you for your comments.

I'm not qualified to comment on the statistical rigor of the analysis, but it seems fine to me. Below are some minor points:

Line 13 Abstract—Typo ‘conducing’

Good catch. We’ve corrected the typo to ‘conducting’ (now, Line 21)

Line 84—Can just use acronym AKP here

We’ve included the acronym in all places that mentioned American Kestrel Partnership after the first mention. Line 28 in the abstract; Line 86 in the body.

Thoughts tangential to this particular paper:
It seems somewhat confounding generally, to try to study the cause of a population decline, while simultaneously prompting people (and lots of them) to create new man-made spaces in the form of nest boxes for these birds to breed. Adding so many nest sites for this species might in itself affect population size, and the outcome of nesting in natural cavities might differ drastically from those in man-made boxes.

We agree with the reviewer and we don’t actually directly encourage people to put up boxes. The AKP’s nuanced perspective is that if people want to help kestrels and they want to put up a box, we serve as a resource for them to obtain the best practices of nest installation, maintenance and monitoring, and data stewardship. It is an interesting position to be in.

6. PLOS authors have the option to publish the peer review history of their article ([what does this mean?](https://journals.plos.org/plosone/s/editorial-and-peer-review-process#loc-peer-review-history)). If published, this will include your full peer review and any attached files.


If you choose “no”, your identity will remain anonymous but your review may still be made public.


**Do you want your identity to be public for this peer review?** For information about this choice, including consent withdrawal, please see our [Privacy Policy](https://www.plos.org/privacy-policy).

Reviewer #1: No

Reviewer #2: No

[NOTE: If reviewer comments were submitted as an attachment file, they will be attached to this email and accessible via the submission site. Please log into your account, locate the manuscript record, and check for the action link "View Attachments". If this link does not appear, there are no attachment files.]

While revising your submission, please upload your figure files to the Preflight Analysis and Conversion Engine (PACE) digital diagnostic tool, <https://pacev2.apexcovantage.com/>. PACE helps ensure that figures meet PLOS requirements. To use PACE, you must first register as a user. Registration is free. Then, login and navigate to the UPLOAD tab, where you will find detailed instructions on how to use the tool. If you encounter any issues or have any questions when using PACE, please email PLOS at [figures@plos.org](mailto:figures@plos.org). Please note that Supporting Information files do not need this step.

*In compliance with data protection regulations, you may request that we remove your personal registration details at any time.* [*(Remove my information/details)*](https://www.editorialmanager.com/pone/login.asp?a=r)*. Please contact the publication office if you have any questions.*

PONE-D-20-40981R1
Evaluating Citizen Science Outreach: A case-study with The Peregrine Fund’s American Kestrel Partnership
Dr Sarah Schulwitz

Dear Dr Schulwitz,

Thank you for submitting your manuscript entitled "Evaluating Citizen Science Outreach: A case-study with The Peregrine Fund’s American Kestrel Partnership" to PLOS ONE.  Your manuscript files have been checked in-house but before we can proceed we need you to address the following issues:

1) Thank you for including your ethics statement on the online submission form: 
"The study was exempt from IRB review due to it being research that included a survey in which responses were obtained in a manner in which the identity of the human subjects was not able to be ascertained directly or through identifiers linked to the respondents. In addition, any disclosure of the responses would not reasonably place the respondents at risk. ".   
To help ensure that the wording of your manuscript is suitable for publication, would you please also add this statement at the beginning of the Methods section of your manuscript file.

Your manuscript has been returned to your account. Please log on to PLOS Editorial Manager at <https://www.editorialmanager.com/pone/> to access your manuscript.

Your manuscript can be found in the "Revisions Sent Back to the Author" link under the New Submissions menu. After you have made the changes requested above, please be sure to view and approve the revised PDF after rebuilding the PDF to complete the resubmission process.

Please note that these changes have been requested to comply with submission guidelines and your manuscript will *not* be sent to review until you have fully adhered to our requests.  Once your paper has been seen by an Editor we may return it to you for further information or amendments.

We ask that you address this request within 21 days. If you require additional time, please email the journal office. We are happy to grant extensions of up to one month past this due date. If we have not heard from you within 21 days, your manuscript will be withdrawn from Editorial Manager.

Thank you for submitting your work to PLOS ONE.

Kind regards,

Anna Fodor
PLOS ONE

****We have added the statement requested at the beginning of the materials section.
